# Effect of Phaco-trabeculectomy versus Phacoemulsification on Refractive Outcome - A Prospective Observational Study

**DOI:** 10.22336/rjo.2025.14

**Published:** 2025

**Authors:** Subashini Kaliaperumal, Krishin K, Mary Stephen, Jayasri P

**Affiliations:** 1 Department of Ophthalmology, Jawaharlal Institute of Postgraduate Medical Education and Research (JIPMER), Puducherry, India; 2 Department of Ophthalmology, Trinity Eye Hospital, Kozhikode, India

**Keywords:** mean prediction error, absolute mean prediction error, phacotrabeculectomy, phacoemulsification, refractive outcomes, IOL = Intraocular Lens, BCVA = Best Corrected Visual Acuity, SE = Spherical Equivalent, D = Diopter, SPSS = Statistical Package for the Social Sciences, SRK-T = Sanders-Retzlaff-Kraff, MMC = Mitomycin C, P = Probability (used in statistical analysis), SD = Standard Deviation, Kolmogorov-Smirnov = A statistical test for the distribution pattern (not an abbreviation but refers to a statistical test)

## Abstract

**Purpose:**

To compare the refractive outcomes of phaco-trabeculectomy versus phacoemulsification.

**Methods:**

This prospective observational study included 75 eyes, 42 eyes with cataract and glaucoma that underwent phaco-trabeculectomy, and 33 eyes with cataract that underwent phacoemulsification. The primary outcome measures were the assessment of mean prediction refractive error and absolute mean prediction refractive error, measured during biometry with a target refraction of more than -1 diopter.

**Results:**

The mean age of the study population was 60.3 ± 4.5 years (SD) in the phaco trabeculectomy group (Group 1) and 64.24 ± 3.2 years (SD) in the phacoemulsification group (Group 2). The mean prediction error in group 1 was -0.21 + 0.88 diopters, and in group 2, it was -0.24 + 1.42 diopters, with absolute mean prediction errors of 0.72 + 0.68 diopters in group 1 and 0.71 + 0.97 diopters in group 2. A statistically significant shift in myopic and hyperopic prediction error was noted for 0 to 1 diopter, and a change above one diopter was not substantial.

**Discussion:**

This discussion examines the challenges associated with refractive outcomes following combined cataract and glaucoma filtration surgery. The study found considerable variability in achieving the target refraction, with prediction errors generally within one diopter, consistent with other research. The study’s limitations, including a short follow-up period and variations in surgical techniques, are acknowledged as possible factors that may contribute to refractive errors and astigmatism.

**Conclusions:**

The predicted refractive errors in both groups were similar, with equal myopic and hyperopic shifts noted, and a statistically significant change was observed from 0 to 1 diopter.

## Introduction

Glaucoma is a relentless, progressive optic neuropathy characterized by distinctive disc changes and corresponding visual field changes, commonly associated with elevated intraocular pressure, but not necessarily so [1]. Trabeculectomy is the conventional treatment for medically uncontrolled glaucoma. But it has been proven time and again to cause cataract in phakic patients [2,3]. The surgery also induces changes in the eye’s biophysical properties that affect the calculation of Intraocular Lens (IOL) power for a subsequent cataract surgery [4]. On the other hand, phacoemulsification in a post-trabeculectomized eye can lead to high bleb failure rates, particularly within the first year [5]. Phacotrabeculectomy has become the surgery of choice in patients with glaucoma not controlled by drugs and visually significant cataract. The advantages of combined procedures include faster postoperative recovery, reduced postoperative intraocular pressure spikes after cataract surgery, and decreased risk of bleb failure associated with subsequent cataract surgery [6]. The disadvantages include early postoperative ocular hypotony and choroidal detachment. Since most of the ambiguity in treating glaucoma stems from guidelines derived from research work and literature, we planned to conduct as much work as possible in this area to draw conclusions that could benefit both surgeons and patients in preoperative planning. Our study aimed to analyze the factors influencing postoperative refraction and predict refractive error to modify biometry calculations preoperatively.

## Methods

This prospective observational study was conducted after obtaining clearance from the Institutional Ethics Committee, and written informed consent was obtained. The purpose and details of the study were explained to the patients in a manner they could understand. The study comprised two groups. Group 1 consisted of patients undergoing phacoemulsification and trabeculectomy, and Group 2 consisted of patients undergoing phacoemulsification only. Patients aged 35-80 who underwent phacotrabeculectomy (Group 1) or phacoemulsification (Group 2) were included. Patients with a poor pre- and/or postoperative visual acuity (<6/60), complications during or after cataract surgery, and those with post-operative refractive surprises were excluded.

Preoperatively, the best-corrected visual acuity (BCVA) and refraction were assessed using a streak retinoscope. Pre-operative biometry was done using the Optical Low Coherence Reflectometry biometer (LENSTAR LS 900 optical biometer, Haag-Streit USA, Mason, Ohio). A qualified and experienced optometrist performed preoperative biometry, and intraocular lens power was selected to give a predicted postoperative refractive error using the SRK-T formula. After taking written and informed consent, phacotrabeculectomy was done in patients planned for a combined surgery. A single-site superior approach without Mitomycin C augmentation was performed in all cases. Cataract surgery was performed using a temporal precise corneal incision phacoemulsification technique in the phacoemulsification group. A foldable hydrophilic acrylic intraocular lens was placed within the capsular bag in both groups. A single surgeon with 15 years of experience in cataract and glaucoma surgeries performed both surgeries.

### Outcome measures

The primary outcome measures were the mean prediction refractive error and the mean absolute prediction error at two months postoperatively. The target refraction aimed for after phacoemulsification and phaco-trabeculectomy was greater than -1 D.

### Sample size calculation

Calculations were performed using OpenEpi software, based on an 80% power and 95% confidence level. Patients were selected using convenience sampling from our outpatient department, undergoing phacoemulsification with intraocular lens implantation.

### Statistical analysis

The analysis was done using SPSS for Windows, Version 20.0. Continuous variables were expressed as mean and discrete variables as proportion. An unpaired Student t-test was used to compare means. A one-sample Kolmogorov-Smirnov test was used to confirm the distribution pattern of the prediction error. All statistical tests were performed at a 5% significance level, and a P value of less than 0.05 was considered statistically significant.

## Results

In our study, 75 eyes were analyzed, comprising 42 in the phacoemulsification group and 33 in the phacoemulsification group. The mean age was 60.3 + 4.5 years (SD) in group 1 and 64.24 + 3.2 years (SD) in group 2. The male: female ratio in group 1 was 2: 1, and in group 2, it was 1: 1. The mean prediction error in group 1 was -0.21 + 0.88 diopter and in group 2 was -0.24 + 1.42 diopter with absolute mean prediction error in group 1 was 0.72 + 0.68 diopter and in group 2 was 0.71 + 0.97 diopter (**Table 1**).

**Table 1:** Demographics and clinical characteristics of patients in both groups

Clinical characteristics	Group 1 (Phacotrabeculectomy) N=42	Group 2 (Phacoemulsification) N=33
Age	60.3 + 4.5 years	64.24 + 3.2 years
Gender		
Male	66.67%	48.49%
Female	33.33%	51.51%
**Pre-operative**		
BCVA		
6/6 - 6/9	6 (14.29%)	4 (12.12%)
6/12- 6/36	24 (57.14%)	24 (72.73%)
6/60	12 (28.57%)	5 (15.15%)
Spherical equivalent in Diopters		
< -1D	11 (26.19%)	7 (21.21%)
-1D to +1D	19 (45.24%)	18 (54.55%)
> +1 D	12 (28.57%)	8 (24.24%)
**Post-operative**		
Post-op BCVA		
6/6 - 6/9	28 (66.67%)	29 (87.88%)
6/12- 6/36	12 (28.57%)	4 (12.12%)
6/60	2 (4.76%)	0
Spherical equivalent (in Diopters)		
< -1D	5 (11.9%)	2 (6.06%)
-1D to +1D	31 (73.81%)	27 (81.81%)
> +1 D	6 (14.29%)	4 (12.13%)

**Graph 1:**
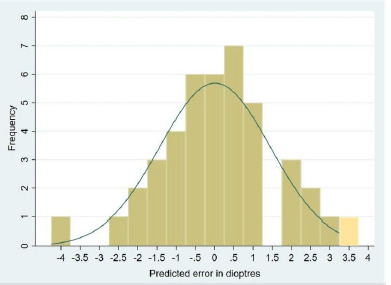
Histogram with Bell curve showing distribution of prediction error among phacotrabeculectomy group (n=42)

**Graph 2:**
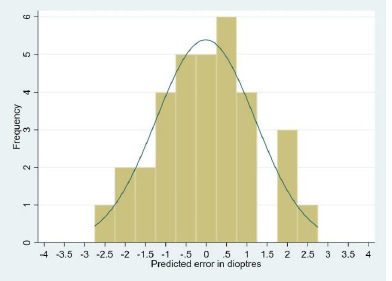
Histogram with Bell curve showing distribution of prediction error among phacoemulsification group (n=33)

The nature of the prediction error was also assessed, and it was found that a myopic or hyperopic shift of 0 to 1 diopter was statistically significant. A shift of more than -1 or more than 1 diopter was not statistically significant, as explained in **Table 2**. A target refraction of -1 diopter was achieved in 31% of Group 1 and 37% of Group 2. The histograms with the distribution curves of prediction error (Graphs 1 and 2) for the cases followed a typical distribution pattern, which was confirmed by the one-sample Kolmogorov-Smirnov test.

**Table 2:** Table representing mean prediction errors in both groups

Data	Phacotrabeculectomy (n=42)	Phacoemulsification (n=33)	P value
SE-PRE	- 0.19 ± 1.5	- 0.18 ± 1.8	0.17
SE-POST	- 0.01 ± 1.1	- 0.14 ± 0.9	0.08
Mean prediction error	-0.21 ± 0.88 Diopter	-0.24 ± 1.42 Diopter	0.28
Absolute mean prediction error	0.72 ± 0.68 Diopter	0.71 ± 0.97 Diopter	0.19
Myopic shift (0 to 1 diopter)	13 (30.95%)	12 (36.36%)	0.02
Myopic shift (>1 diopter)	7 (16.67%)	5 (15.16%)	0.13
Hyperopic shift (0 to 1 diopter)	15 (35.71%)	12 (36.36%)	0.03
Hyperopic shift (> 1 diopter)	7 (16.67%)	4 (12.12%)	0.24

* One sample Kolmogorov-Smirnov Test

## Discussion

Glaucoma is a chronic progressive optic neuropathy that leads to irreversible vision loss. Medical management is beneficial in early cases, but filtration surgery has proven to be very effective in cases not controlled by maximum medical management. Glaucoma is common after 40 years of age in the population where cataracts are also prevalent, and cataract is a common complication post glaucoma filtration surgery. The surgical treatment for cataracts and glaucoma has been refined following the development of small incision surgeries, with the advantages of phacoemulsification including early refractive stabilization and rapid visual recovery. Rockwood et al. evaluated the outcomes of combined cataract extraction, lens implantation, and trabeculectomy surgeries, emphasizing the need for improved refractive predictability in such combined procedures [7]. Their study highlighted that while these surgeries effectively manage both conditions, achieving target refraction remains challenging. Olsen reviewed the calculation of intraocular lens (IOL) power, emphasizing the role of precise biometry in achieving predictable refractive outcomes [8]. Errors in biometry or wound healing can lead to unexpected refractive shifts, which can influence post-surgical vision. Muallem et al. explored the difference between predicted and actual refractive outcomes in cataract surgery performed after trabeculectomy, showing that various factors, including astigmatism and wound healing, impact final refractive results [9]. Tzu et al. conducted a retrospective study on refractive outcomes in combined cataract and glaucoma surgery, reporting that only 74% of cases achieved the intended refraction, highlighting the need for refined surgical techniques [10]. Vaideanu et al. compared one-site phacotrabeculectomy with temporal approach phacoemulsification, revealing differences in refractive outcomes and highlighting the impact of incision location on astigmatism [11]. Murchison and Shields evaluated three different surgical approaches for coexisting cataract and glaucoma. They found that 83% of cases had a prediction error, reinforcing the challenge of obtaining accurate refractive outcomes in combined surgeries [12]. Ong et al. studied a Chinese population undergoing phacotrabeculectomy and phacoemulsification and found that only 27.6% in the phacotrabeculectomy group and 46.2% in the phacoemulsification group achieved the predicted refractive outcome [13]. This study suggested that combined procedures may have less favorable refractive results than sequential surgeries.

Sit et al. analyzed phacoemulsification outcomes using immersion biometry and reported that 84% of patients achieved predicted refractive results, emphasizing the role of precise biometric evaluation in successful refractive outcomes [14]. Finally, Allan and Barrett discussed the advantages of small-incision phacoemulsification combined with trabeculectomy, particularly its benefits in early refractive stabilization and improved visual recovery [15].

Our study findings were comparable, with 31% of participants in Group 1 and 37% in Group 2 achieving the target refraction. The mean prediction error in Group 1 was -0.21 ± 0.88 diopters, and in Group 2 was -0.24 ± 1.42 diopters, with absolute mean prediction errors of 0.72 ± 0.68 diopters and 0.71 ± 0.97 diopters, respectively. A statistically significant shift in myopic and hyperopic prediction errors was observed for deviations of 0 to 1 diopter, whereas changes exceeding one diopter were not statistically significant.

A key limitation of our study is the shorter duration of follow-up. Additionally, phacotrabeculectomy was performed using a single-site superior approach. In contrast, phacoemulsification was performed using a temporal corneal incision, which could be a confounding factor for induced astigmatism in both groups.

## Conclusion

In our study, the refractive outcomes and recovery were similar in the phacotrabeculectomy and phacoemulsification groups, with approximately 35% of participants achieving the target refraction, consistent with other studies. The predicted refractive error in both groups was identical, with equal myopic and hyperopic shifts and a statistically significant change from 0 to 1 diopter.
